# Some Ulcers of the Tongue

**Published:** 1899-08-19

**Authors:** E. Percy Paton

**Affiliations:** Assistant Surgeon to Westminster Hospital, &c.


### Hospital Clinics and Medical Progress.

### SOME ULCERS OF THE TONGUE.

By E. Percy Paton, M.D., M.S., F.R.C.S., Assistant Surgeon to Westminster Hospital, &c.

Ulcers of the tongue may be conveniently classified into the simple, syphilitic, tubercular, and cancerous varieties. The subsequent remarks treat only of the simple variety.

True simple ulcers are commonly dependent upon some source of irritation, such as a jagged tooth, a worn tooth-plate, or a rough pipe mouthpiece, &c.; or they may arise—especially about the sides and tip of the organ—in connection with a condition of dyspepsia. They are also very frequent as a complication of chronic superficial glossitis, owing to the thinness of the epithelial covering of tongue in many of these cases, and consequently its easy destruction by trivial injuries; indeed, this ulceration is one of the main troubles of this form of chronic inflammation of the tongue.

Other forms of ulceration which may be conveniently classified as simple, although, perhaps, not strictly so, are those dependent upon poisons, such as mercury, when administered internally in excessive doses, or occurring amongst workmen who work in this metal, and those due to mycotic or bacterial invasion.

The traumatic ulcer generally presents little difficulty in diagnosis, the cause, if looked for, being usually obvious. One curious variety of it is found in whooping-cough. It is situated on the frænum, and is probably dependent upon the rubbing of this part of the organ against the incisor teeth during the paroxysms of coughing. A traumatic ulcer is usually small in size, somewhat excavated, and with edges which are not raised, nor is there any induration about its base or margins: often it is quite superficial, in which case the edges are commonly somewhat ragged. In cases where a tartar-covered tooth is the cause this frequently exactly fits the ulcer.

The treatment of these ulcers is chiefly to remove the cause. Any tartar-covered tooth should be scaled, or a jagged tooth filed down or removed, and any ill-fitting tooth-plate remedied. When this has been done the ulcer will usually rapidly heal, practically without treatment, but may be assisted by the use of some such mouth-wash as potassii chloratis gr. x., ad aquam ℥i.; or by painting the ulcer with a five-grain to the ounce solution of chromic acid, its surface having been first carefully dried with a small piece of cotton wool. Active caustics, such as nitrate of silver, should not be applied to these ulcers—at any rate in old people—as any powerful irritation has a distinct tendency to maintain the ulceration, and even to cause it to become epitheliomatous; indeed, this possibility in people above middle life should always be kept in mind, and cases which after removal of the cause still continue much as before, more especially if any induration developes about the base of the ulcer, should be looked upon with suspicion, and a small piece at once removed for microscopic examination, after the application of a 10 per cent. solution of cocaine or eucaine to render the procedure painless. By so doing any uneasiness as to the true nature, or tendencies of the case may at once be set at rest; while if the epithelium should be found to have just commenced to implicate the deeper tissues a local removal will rid the patient of a trouble which at a later stage of the disease might not be cured even by a most extensive operation.

The so-called dyspeptic or catarrhal ulcers usually occur about the sides and tip of the tongue; they are red, irritable, and painful, and are often covered by a thin dirty-white fur; sometimes they commence as vesicles which become pustular, and then shed their epithelium. They are usually accompanied by a good deal of pain, and are very irritable. As a rule, they occur in association with some gastro-intestinal disturbance, but this is by no means always the case. Their treatment is simple, except in cases where they occur in connection with great general debility, upon which they are then mainly dependent. Generally speaking, attention to diet and treating the gastric disturbance, if present, in association with some simple mouth wash, such as boracic acid or chlorate of potash, will result in a speedy cure.

Ulcers associated with chronic superficial inflammation of the tongue, and with thickening of the epithelium forming patches of leucoplakia or so-called lingual psoriasis, are by no means so easy to treat—many of the cracks, thin fissures, and superficial excoriations, which occur in these cases, being obstinate to all forms of medication, and when healed recurring again on the slightest provocation. It must also always be borne in mind that it is just these cases in which ultimately the originally simple crack drifts into an epitheliomatous ulcer.

Treatment must in the first instance be directed to rendering the diet as simple and as bland as possible, all hot, spiced, and salted foods must be carefully avoided; this, however, the patient soon learns to do, as the discomfort to which they give rise warns him against them. It is not always so easy to get him to give up alcohol (especially spirits) and smoking; but it is equally necessary. The teeth also must be attended to with scrupulous care, so that any source of irritation may be avoided. Various kinds of local application may then be tried. Weak solutions of chromic acid, namely, from two up to ten grains to the ounce, are most generally useful. The surface of the ulcer is first carefully dried with a piece of cotton wool, and then the lotion is painted on with a brush, this being done once or twice a day, the mouth at the same time being washed out after food with some weak boracic acid lotion.

Other mouth washes that may be used are those containing tincture of myrrh, chlorate of potash, alum; the last, however, should be in very weak solution. The chromic acid above recommended may be gradually increased in strength if the condition does not improve, but it should rarely be used beyond ten grains to the ounce, as it is a stimulating and not an irritating effect which is required. If it is found to be doing no good solutions of sulphate of copper or nitrate of silver in similar strengths may be used in its place. It is often found, however, that this treatment will only keep the condition at bay, so to speak, but will not cure it, and in some cases, indeed, the ulceration remains, in spite of all treatment by lotions, &c., even with the addition, in syphilitic cases, of potassium iodide and mercury administered internally; it then becomes necessary to consider if some benefit cannot be obtained by operation, and this is the more important that it is just in this class of case that epithelioma is so likely to occur on the top of a long-standing irritation. Should the trouble be quite local the best thing that can be done is to remove the portion of the tongue affected; and, as this is mostly situated on its anterior part, it is a matter of little difficulty or risk. When, however, a large area of the organ is affected the operation becomes a severe mutilation, and should only be done in case of absolute necessity, and possibly may be replaced by an operation recommended by Ramsohoff, of Cincinnati, which consists of stripping the whole of the affected portion of the tongue of its mucous membrane, which, he states, can be done with ease and safety, the lost covering being rapidly replaced by epithelium from around the margins of the denuded portion. Of this, however, I have no personal experience.

Ulcers dependent upon the action of poisons, such as mercury, only require the removal of the patient from the influence of the poison, and the use of a simple mouth-wash; but those certainly, or possibly, of mycotic origin, such as occur in thrush or aphthous stomatitis, may require more active treatment. For thrush (due to the oidium albicans or sacchromyces albicans), since the fungus grows well in any sugar-containing medium, this article of diet must be carefully avoided, as also must the usual treatment for this condition by mel boracis. Chlorate of potash, however, may be almost considered a specific for these cases, and should be used both as a mouth-wash, and also administered internally in doses of from five to ten grains or more three times a day, attention being at the same time paid to the diet.

In aphthous stomatitis there is often very extensive ulceration, and much surrounding redness and inflammation, and the patient is in a low general state of health, and to this the trouble is then mostly due; hence measures must be directed towards improving it as far as may be. Mouth-washes of various sorts may also be used, and it is sometimes very advantageous to paint the raw surfaces over with a fairly strong solution of nitrate of silver, chromic acid, or some mercurial solution, such as the perchloride 1-2,000, or the cyanide 1-4,000. At the same time the patient must be moved if possible into airy surroundings, and any possibility of faulty drainage of the dwelling in which the case has occurred must be looked out for and remedied.

